# Supplementary material for: Heparin and Gelatin Co-Functionalized Polyurethane Artificial Blood Vessel for Improving Anticoagulation and Biocompatibility
Source: Bioengineering (Basel). 2025 Mar 18;12(3):304. doi: 10.3390/bioengineering12030304 (PMC11939800; doi:10.3390/bioengineering12030304)
Supplement: Supplementary file 1 [file bioengineering-12-00304-s001.zip › bioengineering-3485171-supplementary.pdf]

bioengineering

MDPI

Article

Heparin and Gelatin Co-Functionalized Polyurethane Artificial Blood Vessel for Improving Anticoagulation and Biocompatibility

Jimin Zhang [1,†], Jingzhe Guo [1,†], Junxian Zhang [1], Danting Li [1], Meihui Zhong [1], Yuxuan Gu [1], Xiaozhe Yan [1] and Pingsheng Huang [2,*]

[1] Hebei Key Laboratory of Functional Polymers, School of Chemical Engineering and Technology, Hebei University of Technology, Tianjin 300130, China; zhangjimin@hebut.edu.cn (J.Z.); 18251836002@163.com (J.G.); 202221501028@stu.hebut.edu.cn (J.Z.); 18830490298@163.com (D.L.); 15864081918@163.com (M.Z.); guyuxuan0a@163.com (Y.G.); 13483017919@163.com (X.Y.)

[2] Tianjin Key Laboratory of Biomaterial Research, Institute of Biomedical Engineering, Chinese Academy of Medical Sciences and Peking Union Medical College, Tianjin 300192, China

* Correspondence: sheng1989.2008@163.com; Tel.: +86-22-15222022066

† These authors contributed equally to this work.

Supplementary materials

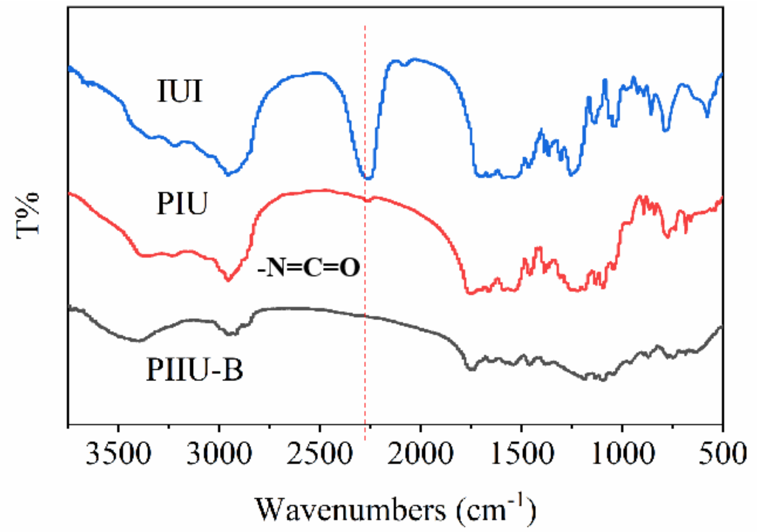

Figure S1. FT-IR spectra of IUI, PIU and PIIU-B.

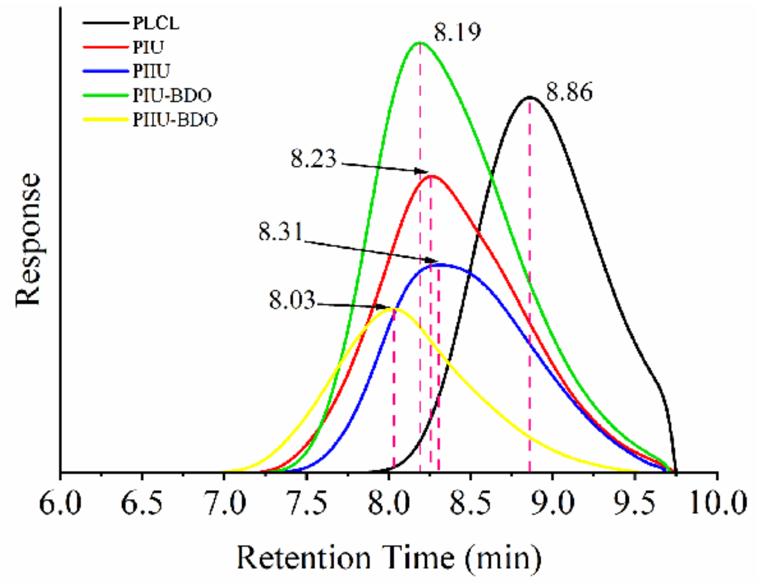

Figure S2. GPC curves of PLCL, PIU, PIIU, PIU-B, PIIU-B.

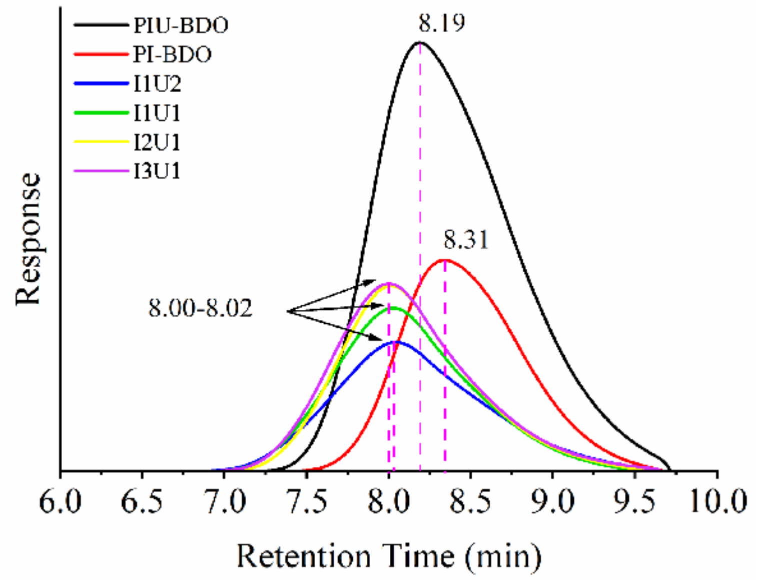

Figure S3. GPC curves of PIIU-B with different IPDI and IUI ratios.

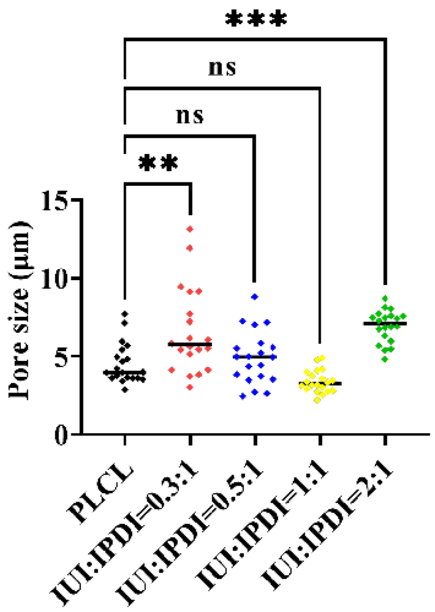

Figure S4. Pore size of PIIU-B with different compositions. Data are presented as mean ± SDs (n = 20), *p < 0.05, **p < 0.01 *** *p* < 0.005, **** *p* < 0.001 Student's t-test.

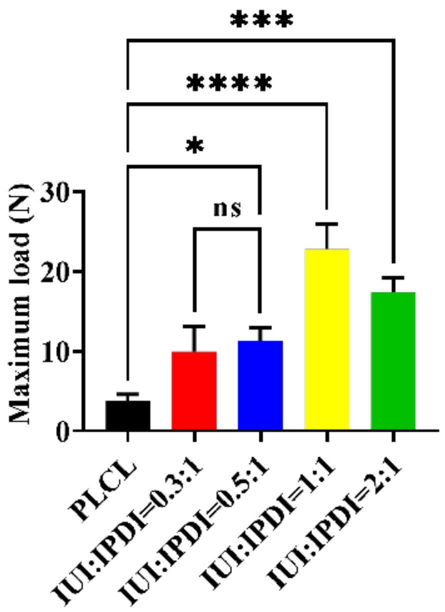

Figure S5. Maximum load of different IPDI and IUI molar ratios (IUI: IPDI=0.3:1, 0.5:1, 1:1, and 2:1). Data are presented as mean ± SDs (n = 3), *p < 0.05, **p < 0.01 *** *p* < 0.005, **** *p* < 0.001 Student's t-test.

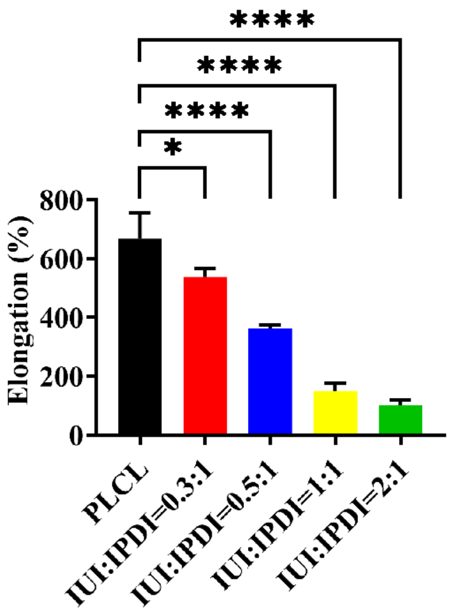

Figure S6. Break longation of PLCL and PIIU-B electrospinning vessles. Data are presented as mean ± SDs (n = 3), *p < 0.05, **p < 0.01 *** *p* < 0.005, **** *p* < 0.001 Student's t-test.

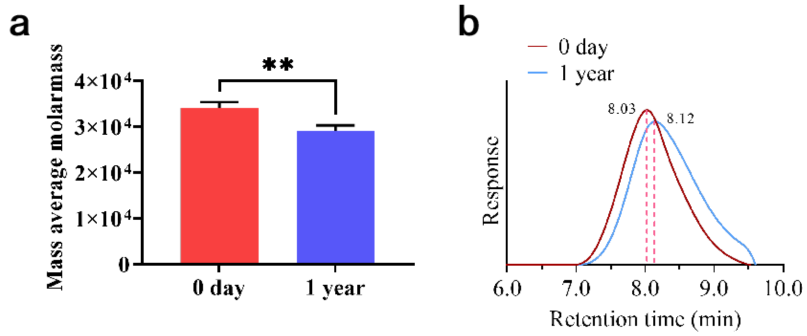

Figure S7. (a) GPC data and (b) GPC curves of PIIU-B (IPDI:IUI=1:1) before and after one-years storage at room temperature. Data are presented as mean ± SDs (n = 3), *p < 0.05, **p < 0.01 Student's t-test.

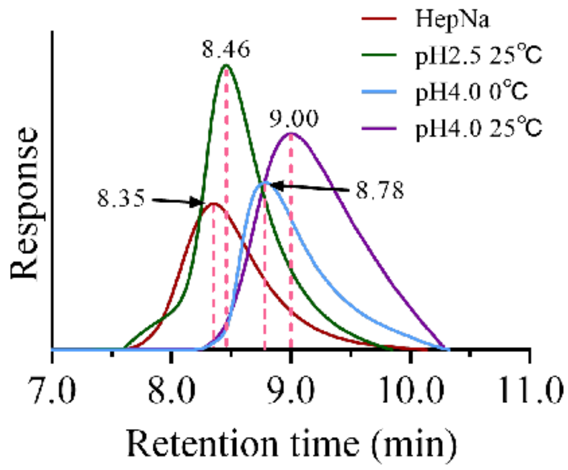

Figure S8. GPC curves of LHepCHO obtained under different reaction conditions.

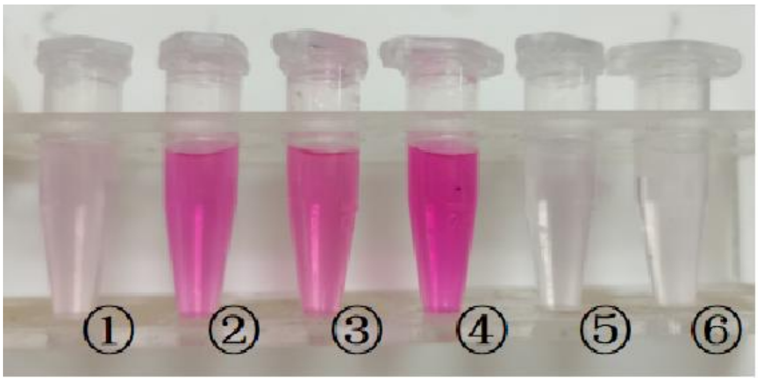

Figure S9. The synthesis of LHepCHO verified by Schiff reagent. ① HepNa, ② -④ LHepCHO (pH4.0/0℃, pH2.5/25℃, pH4.0/25℃), ⑤ LHepMA, ⑥ Control.

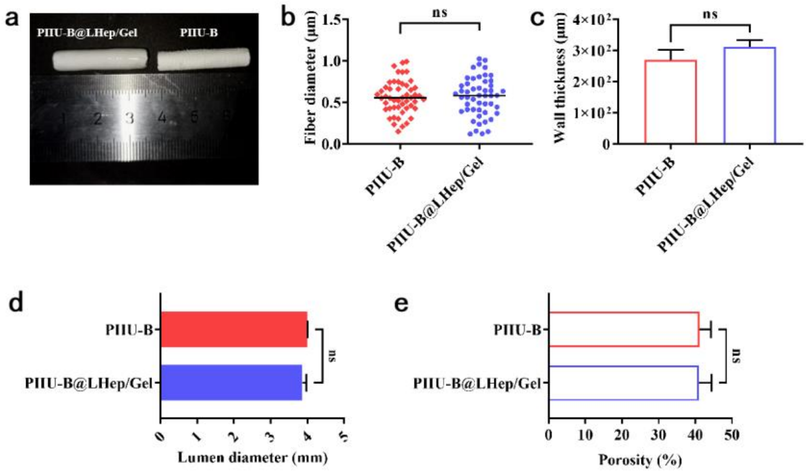

Figure S10. Comparison of PIIU-B with PIIU-B@LHep/Gel after surface functionalization: (a) Optical images, (b) Fiber diameter distribution (n = 50), (c) Wall thickness (n = 3), (d) lumen diameter (n = 3) and (e) porosity (n = 10). Data are presented as mean ± SDs, *p < 0.05, **p < 0.01 Student's t-test.

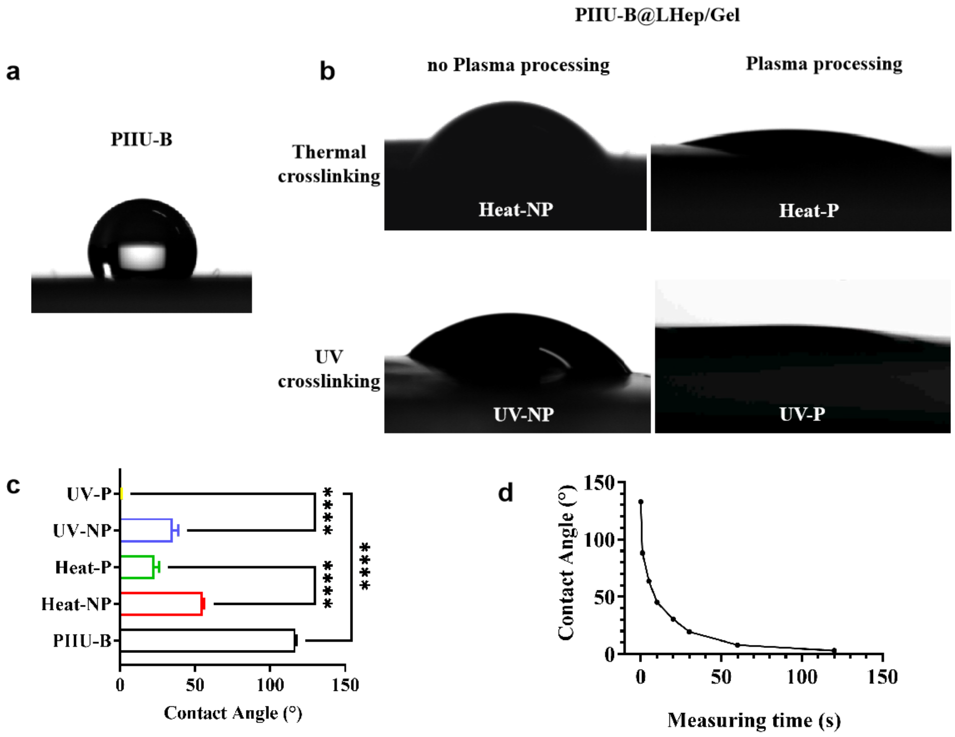

Figure S11. Contact angles of (a) PIIU-B, (b) Contact angles of PIIU-B@LHep/Gel prepared by different crosslinking methods. (c) Statistical analysis of contact angle data. (d) PIIU-B@LHep/Gel contact Angle curve with time. Data are presented as mean ± SDs (n = 3), *p < 0.05, **p < 0.01 Student's t-test.

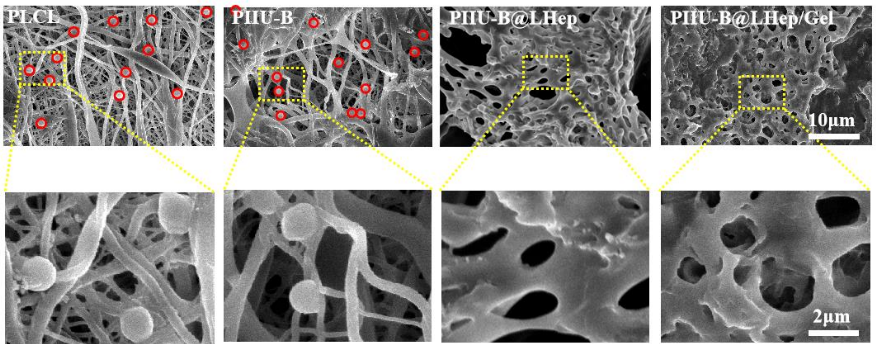

Figure S12. SEM images of platelet adhesion on tubes of PLCL, PIIU-B, PIIU-B@LHep and PIIU-B@LHep/Gel (platelets in the red circle, enlarged images at the bottom).

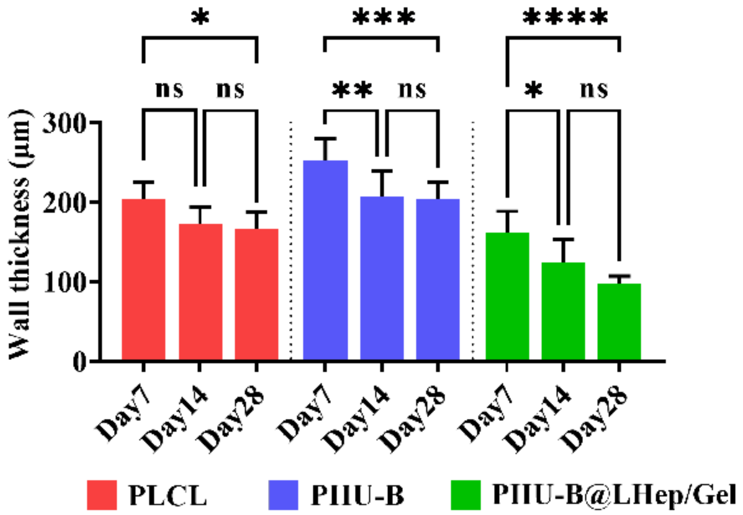

Figure S13. Wall thickness of PLCL, PIIU-B and PIIU-B@LHep/Gel on day0, day7, day14 and day28. Data are presented as mean ± SDs (n = 6), *p < 0.05, **p < 0.01 *** *p* < 0.005, **** *p* < 0.001 Student's t-test.

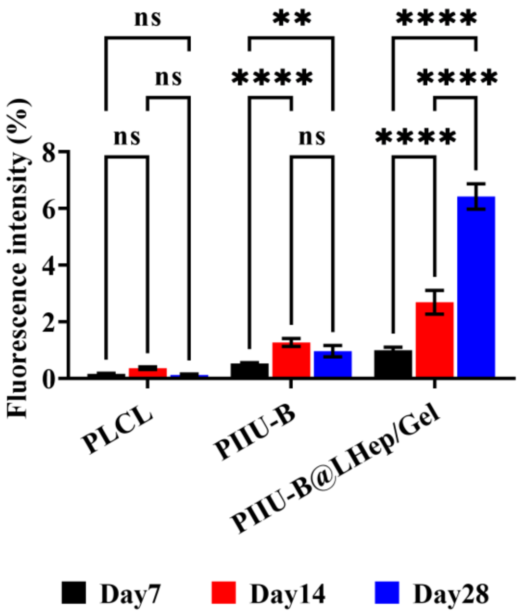

Figure S14. CD68 (red) fluorescence intensity of PLCL, PIIU-B and PIIU-B@LHep/Gel on day0, day7, day14 and day28. Data are presented as mean ± SDs (n = 6), *p < 0.05, **p < 0.01 *** *p* < 0.005, **** *p* < 0.001 Student's t-test.

Disclaimer/Publisher's Note: The statements, opinions and data contained in all publications are solely those of the individual author(s) and contributor(s) and not of MDPI and/or the editor(s). MDPI and/or the editor(s) disclaim responsibility for any injury to people or property resulting from any ideas, methods, instructions or products referred to in the content.
